# Bone regeneration in ceramic scaffolds with variable concentrations of PDRN and rhBMP-2

**DOI:** 10.1038/s41598-021-91147-w

**Published:** 2021-06-01

**Authors:** Ho-Kyung Lim, Yeh-Jin Kwon, Seok-Jin Hong, Hyo-Geun Choi, Sung-Min Chung, Byoung-Eun Yang, Jong-Ho Lee, Soo-Hwan Byun

**Affiliations:** 1 Department of Oral & Maxillofacial Surgery, Korea University Guro Hospital, Seoul, Korea; 2 Department of Otorhinolaryngology-Head & Neck Surgery, Dongtan Sacred Heart Hospital, Hallym University College of Medicine, Dongtan, Korea; 3 Department of Otorhinolaryngology-Head & Neck Surgery, Sacred Heart Hospital, Hallym University College of Medicine, Anyang, Korea; 4 R&D Center, Genoss, Suwon, Korea; 5 Department of Oral & Maxillofacial Surgery, Sacred Heart Hospital, Hallym University College of Medicine, Anyang, Korea; 6 Graduate School of Clinical Dentistry, Hallym University, Chuncheon, Korea; 7 Department of Oral & Maxillofacial Surgery, School of Dentistry, Seoul National University, Seoul, Korea

**Keywords:** Dental diseases, Oral diseases

## Abstract

This study evaluated the bone regeneration capacity and mechanical properties of block-type hydroxyapatite (HA)/tricalcium phosphate (TCP) scaffolds in response to different concentrations of polydeoxyribonucleotide (PDRN) and recombinant human bone morphogenic protein 2 (rhBMP-2). Thirty-two male white rabbits were used as a model of calvarial bone defect and classified into eight groups according to type and concentration of growth factor administered, viz., control group (only HA/TCP scaffold), scaffold + PDRN (0.1, 1, 5, and 10 mg/mL each) and scaffold + rhBMP-2 (0.01, 0.05, and 0.1 mg/mL each). The specimens were evaluated using histomorphometric and radiological analyses. Histomorphometric analyses indicated that the administration of PDRN did not increase bone formation. However, significant increases in bone formation were observed with the administration of rhBMP-2 at 0.05 and 0.10 mg/mL on week 8 compared to the control (*p* < 0.05). Radiological analyses revealed a significant increase in bone formation at week 8 with the administration of PDRN at 5 mg/mL and 10 mg/mL, and rhBMP-2 at 0.05 or 0.10 mg/mL compared to the control (*p* < 0.05). Our findings show that block-type HA/TCP scaffolds possess sufficient mechanical strength and bone regeneration capacity when used with optimal concentrations of growth factors.

## Introduction

Bone grafting in the oral and maxillofacial area to correct bony defects caused by pathology, trauma, or aging has been successful clinically^1^. Bone grafts are classified into autogenous, allogeneic, xenogeneic, and alloplastic bone grafts according to the tissue origin of the graft^2^; among these, autogenous bone graft has the advantage of being rich in several osteogenic factors but carries the disadvantage of a surgical wound being created at the donor site^3^. Allogenic and xenogeneic bone grafts are considered disadvantageous due to ethical constraints and the possibility of immune reactions^4^. Most of the commercially available bone graft materials, such as onlay or veneer type grafts, exist in powder form and can completely fill the defects in the bone. However, bone powder is difficult to use in mechanically supported areas^5^. Therefore, block bone harvesting using autogenous bones collected from the iliac, ramus, and chin has been used for bone grafts that require mechanical support^6^. However, autogenous bone collection has several disadvantages, such as longer operation time, discomfort at the donor site, and re-operation challenges if the bone graft fails^7^. The use of synthetic block-type bones that can replace autogenic block bone harvesting has limited clinical applications due to the lack of bone formation ability and the insufficient mechanical strength of large block sizes^8^. On the other hand, alloplastic bone grafts are free from the shortcomings related to the supply of autogenous, allogeneic, and xenogeneic bone grafts but are deficient in bone formation capability^9^. Therefore, to overcome the limitations of bone formation capability, many clinical trials have been conducted on bone grafting using alloplastic bone grafts along with growth factors^10^.

Currently, bone morphogenic proteins (BMPs) are the most studied growth factors in bone regeneration^11^; among them, recombinant human bone morphogenic protein 2 (rhBMP-2), which belongs to the transforming growth factor-β superfamily, is obtained from mammalian cells by recombinant DNA technology and shows the best bone induction ability^12^. Using gene cloning, Wozney et al. purified rhBMP-2 and confirmed that BMP belongs to the transforming growth factor-β family^13^. In 2007, the United States Food and Drug Administration approved the use of rhBMP-2 in the oral and maxillofacial regions for sinus floor augmentation and extraction socket preservation^14^. Most studies have documented consistent findings regarding the osteoinductive ability of rhBMP-2, but a few studies reported that it could induce malignant tumors and even inhibit bone formation at high concentrations^15,16^.

Polydeoxyribonucleotide (PDRN) is a growth factor that has been studied in soft tissue healing and is effective in skin regeneration, angiogenesis, and wound repair^17,18^. PDRN is a mixture of deoxyribonucleotides derived from *Oncorhynchus mykiss* (Salmon trout) and *O. keta* (Chum Salmon) sperm DNA^19^. PDRN exhibits various therapeutic effects by promoting the expression of vascular endothelial growth factor that regulates angiogenesis^20^. PDRN promotes rapid re-epithelialization of deep-dermal burn injury^21^, increases the survival rate of skin random flap^22^, promotes re-epithelialization in free skin graft^23^, accelerates regeneration of corneal epithelial cells^24^, and increases therapeutic effects in diabetic foot ulcers^25^ and pressure ulcers^26^. As such, PDRN is an excellent growth factor for soft tissue treatment. Although the bone regeneration capacity of PDRN has not been studied extensively, a few reports have suggested that PDRN promotes osteoblast differentiation and has bone-inducing properties^19,27^.

In this study, a block-type scaffold composed of hydroxyapatite (HA) and tricalcium phosphate (TCP) was fabricated, and its mechanical strength was measured to investigate the clinical applicability of block-type ceramic scaffold grafts. In addition, various concentrations of rhBMP-2 and PDRN were used to compensate for the insufficient bone-inducing ability of block-type ceramic scaffold grafts, and the optimal concentrations of rhBMP-2 and PDRN were determined to maximize new bone formation. The prospective significance of this study is to provide proper concentration of rhBMP-2 and PDRN for clinicians.

## Results

### Compressive strength of HA/TCP scaffold block

The compressive strength of HA/TCP scaffold blocks was calculated to be 6.76 ± 1.66 MPa.

### Clinical findings in animal experiments

Specific clinical signs of death were not observed in all the animals during the observational period. Additionally, there were no apparent abnormal symptoms of infection or inflammation on the operated sites. No abnormalities in the skull shape or unusual lesions were observed at the time of euthanasia on the 4th and 8th weeks post-operation.

### Radiological analysis

**Figure 1:**
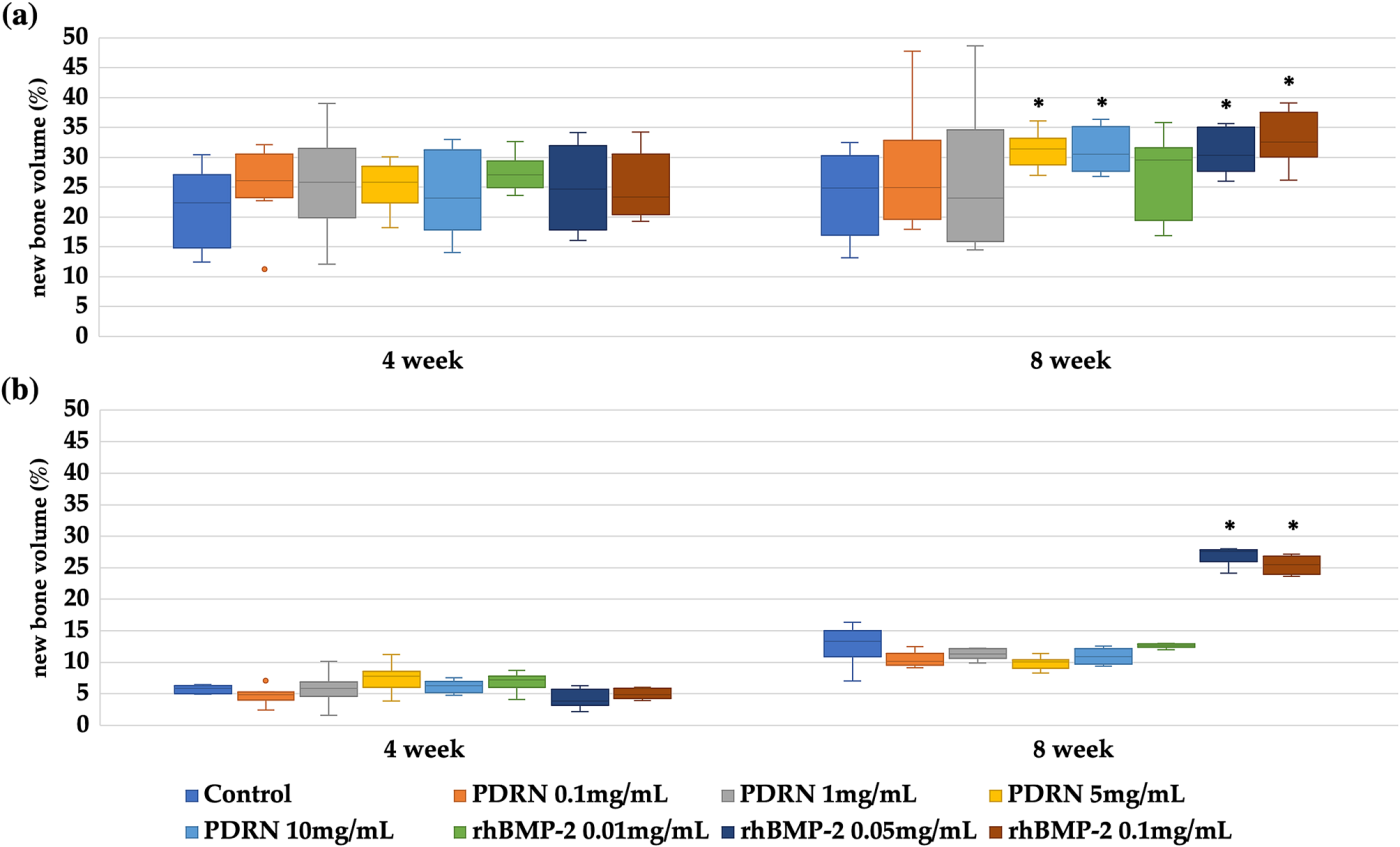
New bone formation in response to HA/TCP scaffolds with variable concentrations of PDRN and rhBMP-2 (*n* = 16, for each). (**a**) Radiological analysis; (**b**) histomorphometric analysis; *statistically significant (*p* < 0.05); box plot, 1st/3rd quartile; *PDRN* polydeoxyribonucleotide; *rhBMP-2* recombinant human bone morphogenic protein.

**Figure 2:**
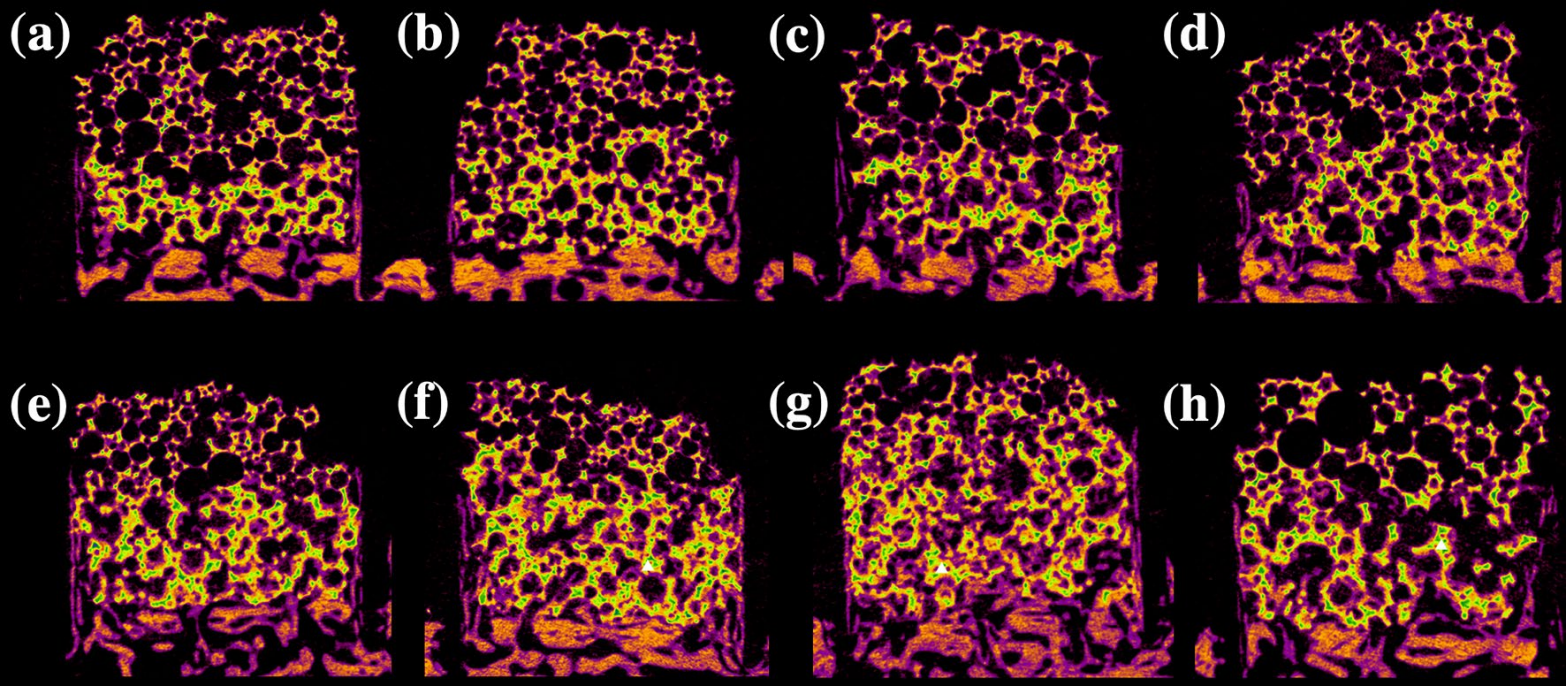
Radiological analysis of new bone formation in response to HA/TCP scaffolds in New Zealand white rabbits 8 weeks post-operation.; (**a**) 0.1 mg/mL PDRN; (**b**) 1.0 mg/mL PDRN; (**c**) 5 mg/mL PDRN; (**d**) 10 mg/mL PDRN; (**e**) 0.01 mg/mL rhBMP-2; (**f**) 0.05 mg/mL rhBMP-2; (**g**) 0.1 mg/mL rhBMP-2; (**h**) Control group; (white triangle) new bone. *PDRN* polydeoxyribonucleotide; *rhBMP-2* recombinant human bone morphogenic protein; *HA* hydroxyapatite; *TCP* tricalcium phosphate.

At 4 weeks post-operation, micro-computed tomography (μCT) scans revealed no significant differences in new bone formation in the animals of the group administered with PDRN and the control group (*p* > 0.05). However, at 8 weeks post-operation, new bone formation in the groups administered with 5 mg/mL and 10 mg/mL PDRN was significantly more than that in the control group (*p* < 0.05) (Figs. 1 and 2). Additionally, there was no significant difference in bone formation in the group treated with 5 mg/mL PDRN compared to that in the group treated with 10 mg/mL PDRN (*p* > 0.05) (Figs. 1 and 2).

At 4 weeks post-operation, μCT scans showed no significant difference in bone formation between the experimental groups administered with rhBMP-2 and the control group. However, at 8 weeks post-operation, new bone formation was significantly higher in the groups administered with 0.05 and 0.1 mg/mL rhBMP-2 compared to that in the control group (*p* < 0.05). The extent of bone formation differed significantly in the groups administered with 0.05 and 0.1 mg/mL of rhBMP-2, and the extent of new bone formation increased at higher concentrations (*p* < 0.05) (Figs. 1 and 2).

### Histomorphometric analysis

**Figure 3:**
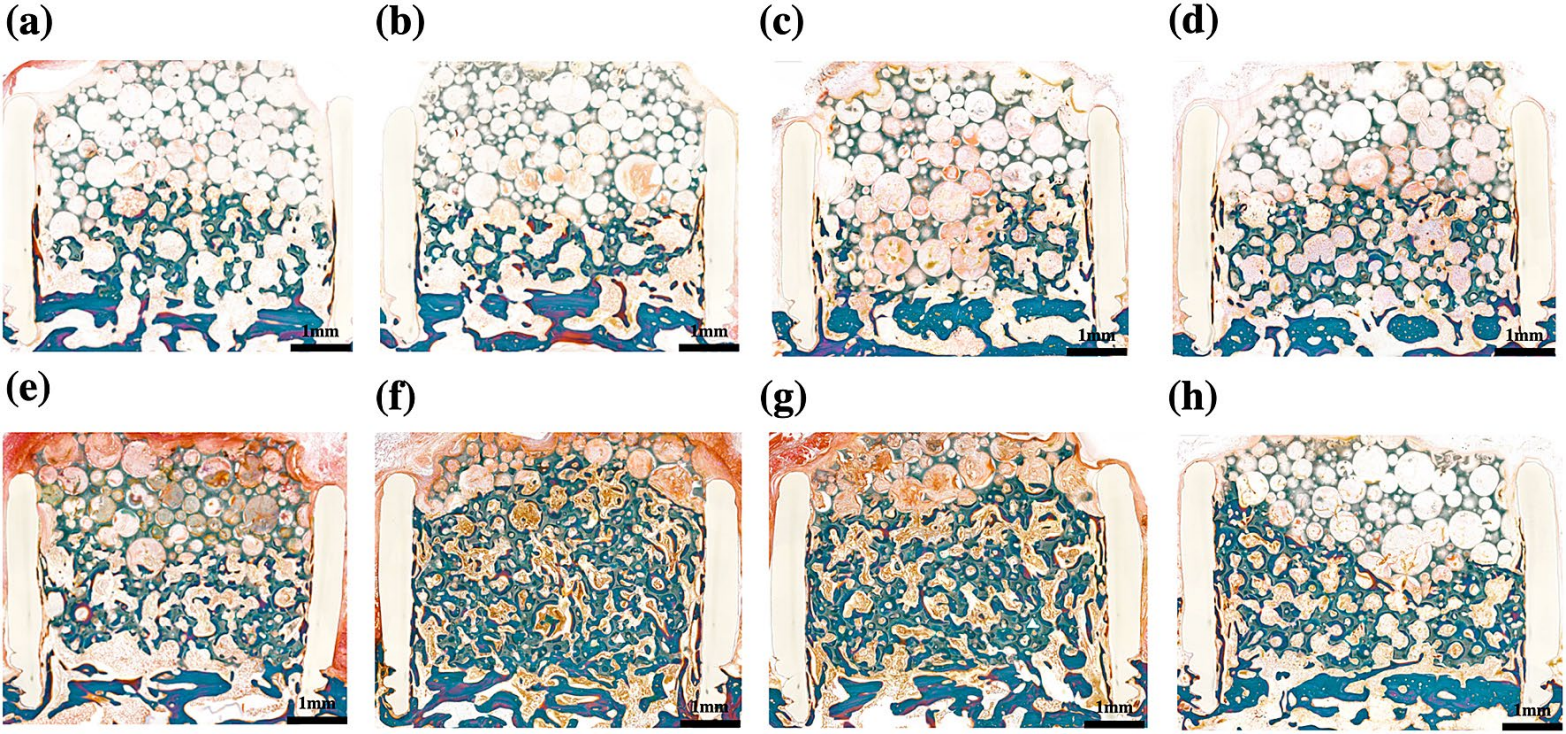
Histomorphometric analysis of new bone formation in response to HA/TCP scaffolds in New Zealand white rabbits 8 weeks post-operation. (**a**) 0.1 mg/mL PDRN; (**b**) 1.0 mg/mL PDRN; (**c**) 5 mg/mL PDRN; (**d**) 10 mg/mL PDRN; (**e**) 0.01 mg/mL rhBMP-2; (**f**) 0.05 mg/mL rhBMP-2; (**g**) 0.1 mg/mL rhBMP-2; (**h**) Control group; (white triangle) new bone. *PDRN* polydeoxyribonucleotide; *rhBMP-2* recombinant human bone morphogenic protein; *HA* hydroxyapatite; *TCP* tricalcium phosphate.

Histomorphometric analyses of the specimens revealed no significant differences in new bone formation between the experimental groups administered with PDRN and the control group at both 4 and 8 weeks post-operation (*p* > 0.05) (Figs. 1 and 3). Similarly, the group administered with rhBMP-2 did not significantly differ in new bone formation compared to the control group at 4 weeks post-operation (*p* > 0.05). However, at 8 weeks post-operation, experimental groups administered with 0.05 and 0.1 mg/mL of rhBMP-2 showed a significant increase in new bone formation compared to the control group (*p* < 0.05) (Figs. 1 and 3). Additionally, there was no difference in the extent of bone formation between the groups administered with 0.05 and 0.1 mg/mL rhBMP-2 (*p* > 0.05) (Fig. 3).

## Discussion

The compressive strength of the HA/TCP block used in the study was approximately 6.0 MPa. Various studies have reported the mechanical strength of human bone. In one study, the ultimate compressive strength of normal human trabecular bone from the mandible was reported to range from 0.22 to 10.44 MPa, with a mean value of 3.9 MPa (SD = 2.7)^28^. Another report suggested that the mean values of compressive strength for the anterior, premolar, and molar region bone specimens obtained from six embalmed human cadaver mandibles were 23.7, 21.6, and 19.6 MPa, respectively^29^. In a study using finite element analysis, the mean ultimate compressive strength of the mandible was found to be 19.73 MPa (range, 0.22 to 10.44 MPa)^30^. In an investigation carried out to study human anterior iliac crests, the mean ultimate compressive strength was reported to be 13.58 MPa (range, 2.43 to 16.75 MPa)^31^. The HA/TCP block used in this study showed higher compressive strength than the trabecular bone in the mandible and marginally lower compressive strength than the whole mandible or iliac bone. Therefore, HA/TCP block is adequately robust for clinical use.

Previous studies have shown that rhBMP-2 induces clinically acceptable bone formation in multiple craniofacial areas and periodontal sites in animal and human models^32,33^. Due to the easy solubility of rhBMP-2 when used in isolation, HA or β-TCP could be used as delivery carriers because of their space-filling nature^34^. Earlier studies used powdered ceramic as a carrier for supporting rhBMP-2^12,13^. Additionally, a recent study reported the generation of pure ceramic blocks using 3D printers^35^. Recent studies have also shown promising results using block-type ceramic as rhBMP-2 carrier^36,37^. In line with these studies, Misch et al. reported that when the allogenic block was covered with rhBMP-2 and collagen sponge and assessed for 6 months after engraftment into the human maxilla, the block volume decreased by an average of 14.7%, and the implant placed at that location had a survival rate of 98.7%^36^. Lee et al. reported that the volume of the allogenic block inserted into rabbit calvaria was better maintained compared to the control group when the block was soaked with 0.1 mg/mL rhBMP-2^37^.

The osteoinductive ability of rhBMP-2 has been validated by several studies, and rhBMP-2 is known to induce excellent bone formation regardless of the type of bone graft used^33,38^. However, some variations have been observed for each type of graft material. In a study conducted using a rat model system, where autogenic, allogeneic, and xenogeneic bones were grafted with 0.05 mg/mL rhBMP-2, higher osteoclastic markers such as tartrate-resistant acid phosphatase and type 2 and 9 metalloproteinases were observed in the xenogeneic group^39^. In another study with a similar design, osteoblastic markers such as osteocalcin, bone sialoprotein, and vascular endothelial growth factor were higher in the autogenic and allogenic groups^40^. In a study comparing rhBMP-2 administration to xenogeneic and alloplastic bones, slow release of rhBMP-2 was observed in the xenogeneic group^40^. A study comparing TCP and HA with the scaffold of rhBMP-2 reported that regarding volume maintenance for graft material, TCP was a more effective carrier than HA^41^. Although further research on rhBMP-2 and bone graft material is warranted, the results of the above studies suggested that while autografts and allografts may be effective in relation to bone formation markers, xenografts are effective in relation to the slow release of rhBMP-2, and TCP are more effective than HA in relation to volume maintenance.

The optimal concentration of rhBMP-2 for bone induction has not been established to date. In a clinical study comparing a placebo with 0.75 mg/mL and 1.50 mg/mL rhBMP-2 for socket preservation, new bone generation was significantly higher in response to treatment with 1.50 mg/mL rhBMP-2^42^. An animal study conducted on pigs suggested that 120 μg/mL rhBMP-2 was effective for the regeneration of mandibular defects^43^. A study reported the use of rhBMP-2 in canines. The study found that the concentration of BMP-2 delivered affected the healing efficacy, with higher concentrations displaying more osteogenesis and a more pronounced bone regeneration^44^. Previous studies were not performed under sealed conditions. rhBMP-2 is mostly delivered as a fluid-type, so it can be dispersed around tissues. Taking a different perspective, this study used a customized polycarbonate tube to prevent the dispersion of rhBMP-2. In our study, the 0.05 mg/mL and 0.1 mg/mL rhBMP-2 groups showed better bone induction compared to the control and 0.01 mg/mL rhBMP-2 groups. Thus, the outcomes of the present study provide novel data, which further add to the existing literature^42,43^. Although the present study investigated bone regeneration using various concentrations of rhBMP-2 and PDRN in multiple specimens, their ability to increase bone formation may differ between animal and clinical studies due to the differences in body weight among subjects^45^.

Several clinical complications may occur post engraftment, depending on the concentration of rhBMP-2 administered, including the suppression of bone formation. A study showed that when calcium phosphate soaked in more than 1.5 mg/mL of rhBMP-2 was grafted for sinus augmentation, bone formation was locally suppressed^46^. Although BMP induces bone formation, it plays a multifunctional role in bone development, such as affecting the osteoclastic bone-resorbing activity^15^. Another clinical complication associated with the administration of a high concentration of rhBMP-2 is the development of malignancies in the subject. A report indicated that the differentiation of oral squamous cell carcinoma cells was promoted when the concentration of rhBMP-2 was ≥ 5 ng/mL^47^. In addition, studies have shown that lung and breast cancer were promoted in subjects at an rhBMP-2 concentration of 2 μg/mL and ≥ 10 ng/mL, respectively^48,49^. Apart from the challenges described above, administration of zoledronate, a type of bisphosphonate, disturbed the osseointegration of the implant at higher concentrations of rhBMP-2 and led to bone erosion around the implant^50^. Therefore, it is essential to administer rhBMP-2 at an optimal concentration to enhance bone formation.

Few studies have suggested that PDRN can be an effective growth factor for bone regeneration as well as soft tissue. Induction in osteoblast growth with a concomitant increase in alkaline phosphatase after 6 days was observed at a concentration of 1 mg/mL PDRN compared with that in the control group^19^. When 10 μg/mL of PDRN was administered to mice, osteoblast differentiation and new bone formation were significantly increased in the second week post-treatment^27^. In the radiological analysis in the present study, increment in bone formation was observed at PDRN concentrations of 5 mg/mL and 10 mg/mL at 8 weeks, and no further increase in bone formation was observed upon increasing the concentration. However, histomorphometric analysis did not reveal any significant differences among the PDRN groups. To establish PDRN as a reliable growth factor for bone regeneration like rhBMP-2, more studies are warranted to determine the appropriate PDRN dose and its effect on bone formation.

## Conclusions

Successful bone induction without specific complications was observed in this study using HA/TCP block graft treated with rhBMP2 and PDRN. HA/TCP blocks showed adequate compressive strength for clinical use. A significant increase in bone formation was observed, both radiologically and histomorphometrically, when the rhBMP-2 concentration was 0.05 and 0.1 mg/mL. Thus, our research findings indicate that the block type HA/TCP scaffolds have adequate compressive strength for clinical usage and show significant bone regeneration capacity when used with an optimal concentration of rhBMP-2. Further studies are now needed to investigate the efficacy of PDRN for bone regeneration and elucidate the underlying mechanisms to maximize the advantages of using rhBMP-2 and PDRN simultaneously.

## Materials and methods

### Manufacture of HA/TCP scaffold block

A synthetic bone graft material (OSTEON 3 BLOCK, GENOSS, Suwon, Korea) consisting of HA and β-TCP at a ratio of 60:40 was prepared. The Ca/P ratio of the synthetic bone graft material ranged from 1.5 to 2.0. The biphasic scaffold was fabricated using the direct foaming method. Briefly, a foamed ceramic material was made from ceramic precursors and additives, and then it was dried and sintered. The specifications of the produced scaffold were as follows: outer dimension was 7 mm diameter × 5 mm height, porosity was approximately 80%, pore size distribution was 200 ~ 400 μm, pore structure was mostly open porosity, and closed porosity was less than 3%.

### Compression test of HA/TCP scaffold block

The compressive strength of the HA/TCP scaffold block was evaluated using the standard test method for compressive properties of rigid plastic. Block-type specimens of dimensions 10 × 10 × 10 mm^3^ were prepared. After placing the specimen on the plate, the rod of the universal testing machine (H50K-T UTM, TINIUS OLSEN Corp., Surrey, UK) was maneuvered in the downward direction, at a rate of 0.5 ± 0.1 mm/min. Thereafter, the load applied to the specimen was measured automatically by a computer program. The derived load data was processed and calculated using the cross-sectional area of the specimen. The distance (μm, linear scale) was plotted against the stress [(N/cm^2^), log scale] value. The compressive stress values were extracted from the graph by finding the intersection of the regions where the stress increased rapidly and where the stress increased gradually. The compressive value was measured at the time of translation from elastic deformation to plastic deformation. A total of 30 block-type specimens were used for the compression test.

### Animal experiments

**Figure 4:**
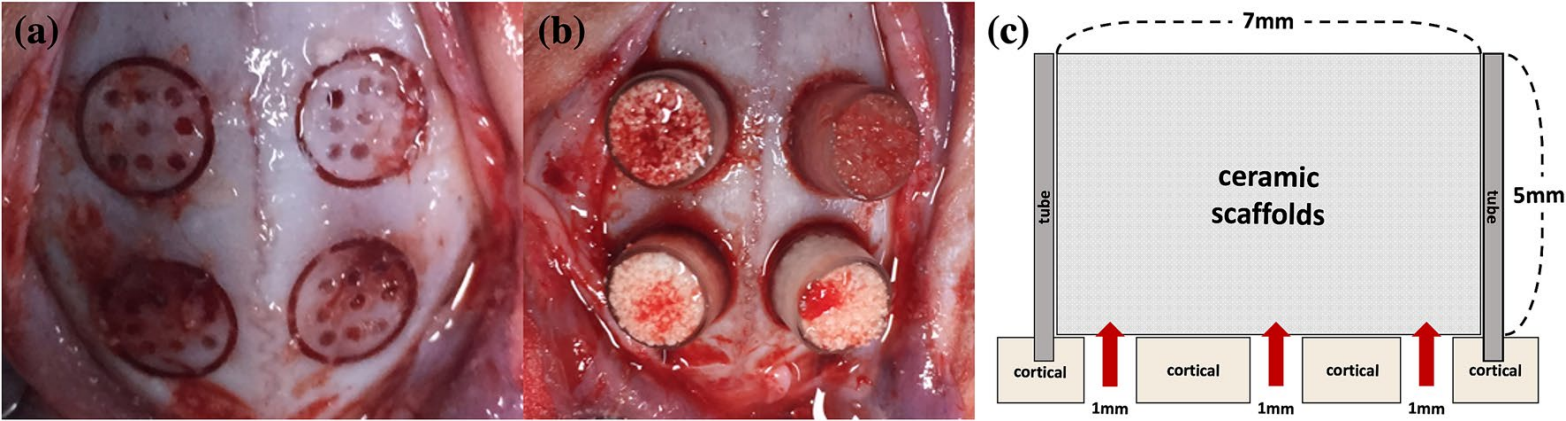
Subperiosteal dissection of the calvarium of New Zealand white rabbits. **(a)** Four borders on each calvarial bone. Each 7 mm diameter circular border contained nine holes of 1 mm diameter; **(b)** Insertion of scaffold into customized polycarbonate tubes (Φ 7 × 5 mm) **(c)** Diagram of experimental model.

Thirty-two seven-week-old New Zealand white rabbits (weight, 2.6 ± 0.3 kg) were classified into eight groups according to the type and concentration of growth factor administered, viz., control group (only HA/TCP scaffold), scaffold + PDRN (0.1 mg/mL, 1 mg/mL, 5 mg/mL, and 10 mg/mL) and scaffold + rhBMP-2 (0.01 mg/mL, 0.05 mg/mL, and 0.1 mg/mL). General anesthesia was provided by intramuscular administration of a combination of tiletamine and zolazepam (ZOLETIL, 50 mg/kg, VIRBAC KOREA, Seoul, Korea) and xylazine HCl (ROMPUN, 10 mg/kg, BAYER KOREA, Seoul, Korea). After cleaning with betadine and shaving the surgical site, 1:100,000 epinephrine was administered subcutaneously for hemostasis. An incision was made, and subperiosteal dissection was performed on the forehead. After the bone was exposed, preparations for the bone augmentation model were performed. Four round-shaped borders of 7-mm diameter were designed and drawn on the calvarial bone. Additional nine holes of 1-mm diameter were formed within each round border for enhancing bone regeneration capacity and blood supply. Prefabricated polycarbonate tubes (Φ 7 mm diameter × 5 mm height) were fitted into the 7-mm round borders, and the block-type ceramic scaffolds were designed and inserted into the tubes^51^ (Fig. 4). PDRN solution (PDRN, GENOSS, Suwon, Korea) and rhBMP-2 solution (BMP, GENOSS, Suwon, Korea) at different concentrations (up to a total of 0.1 mL volume), were filled into the tubes. After covering the tubes with a lid to prevent sample leakage, a continuous suture was made using 4–0 VICRYL (JOHNSON & JOHNSON, Brunswick, NJ, USA). Gentamicin sulfate (5 mg/kg, intramuscular, SINIL, Seoul, Korea) and diclofenac (5 mg/kg, IM, SINIL, Seoul, Korea) were administered post-operatively for 3 days for their antibiotic and analgesic effects, respectively. The post-operative monitoring of rabbits was performed weekly for inflammation, infection, wound dehiscence, and general health until they were sacrificed. Eight animals were sacrificed on the 4th- and 8th-week post-operation. After anesthesia using a combination of tiletamine, zolazepam, and xylazine hydrogen chloride, the animals were euthanized by administering potassium chloride to the marginal ear vein. After euthanasia, the skull was dissected, separated, and fixed with 10% formalin solution.

All experiments were approved by the Genoss Laboratory Animal Ethics Committee based on the law of laboratory animals (Approval No: GEN-IACUC-1908–02). These animal experiments were conducted according to the ARRIVE (Animal Research: Reporting of In Vivo Experiments) guidelines. All methods were carried out under relevant guidelines and regulations.

### Radiological analysis

The extracted skulls were studied using a μCT machine (SKYSCAN1173, Ver. 1.6, BRUKER-CT, KONTICH, Belgium) for imaging. The pre-set imaging conditions were 60 μA tube current, 130 kVp tube voltage, 500 ms exposure time, 1 mm aluminum filter, and 0.3° rotation angle. The pixel size was 13.85 μm, and the number of pixels was 2240 × 2240. With the help of a μCT scan, a total of 800 raw high-resolution images were obtained. The NRecon program (Ver 1.7.0.5, BRUKER-CT, KONTICH, Belgium) was used for the cross-sectional reconfiguration. The Dataviewer program (Ver. 1.5.1.3, BRUKER-CT, KONTICH, Belgium) and the Ct-VOX program (Ver. 1.14.4.2, BRUKER-CT, KONTICH, Belgium) were used for 3D reconstruction. The volume of newly formed bone inside the scaffolds was calculated using the difference in grayscale level. In the program, since the HU (Housefield unit) of the scaffold was 400, the HU of the cortical bone was 600 or more, and that of the soft tissue was 100 or less, the grayscale threshold of the new bone was set to 150–350 HU. Newly formed bone volume in the scaffolds was calculated using these programs, according to the following calculation:

1 $$ {\text{Percent}}\;{\text{bone}}\;{\text{volume}}\;\left( \%  \right) = {\text{New}}\;{\text{bone}}\;{\text{volume}}/{\text{Total}}\;{\text{volume}}\;{\text{in}}\;{\text{scaffold}} \times 100  $$

### Histological analysis

After incubating the samples in formalin for 1 week, the extricated specimens were rinsed with flowing water for 9 ± 3 h and then trimmed for further processing. Undecalcified preparation of bone tissue was performed. The specimens were dehydrated with a graded ethanol series of 70%, 95%, and 100%. The tissue samples were then infiltrated with a solution of alcohol and Technovit 7200 Resin (HERAEUS KULZER, Hanau, Germany) with a subsequent increase in resin ratio in the solution. Finally, penetration of Technovit 7200 resin was performed by vacuuming the stock solution for 2 days. The resin was cured using a UV Embedding system (KULZER EXAKT 520, HERAEUS KULZER, Hanau, Germany), and the formatted resin block was cut using an EXAKT diamond cutter (KULZER EXAKT 300, HERAEUS KULZER, Hanau, Germany) to obtain a cross-section of the extracted specimens. The initial section had a thickness of 300 ± 50 μm, and the section was then polished using an EXAKT grinding machine (KULZER EXAKT 400CS, HERAEUS KULZER, Hanau, Germany) until the desired thickness of 40 ± 5 μm was achieved. Thin tissue sections were stained using Goldner’s trichrome. The images of the tissue slides were obtained using an optical microscope (OLYMPUS BX50, OLYMPUS OPTICAL Co., Tokyo, Japan) with a charge-coupled device camera, at magnifications of × 1.25, × 4, × 10, and × 20. Subsequently, the percentage of the new bone was calculated using IMAGE-PRO PLUS software (MEDIA CYBERNETICS, Rockville, MD, USA) as follows:

2 $$ {\text{Percent}}\;{\text{New}}\;{\text{bone}}\;{\text{area}}\;\left( \% \right) = {\text{Area}}\;{\text{of}}\;{\text{new}}\;{\text{bone}}/{\text{Area}}\;{\text{of}}\;{\text{total}}\;{\text{in}}\;{\text{scaffold}} \times {1}00 $$

### Statistical analysis

The Kruskal–Wallis test was used to analyze the differences in bone regeneration capacity among the groups treated with different concentrations of rhBMP-2 and PDRN. Mann–Whitney U tests were used to analyze the comparisons between the control and experimental groups. The significance level was set at 0.05, and statistical analyses were performed using the SPSS program (version 20, SPSS Inc., Chicago, IL, USA).
